# Isolated avulsion of the vastus lateralis tendon insertion in a weightlifter: a case report

**DOI:** 10.4076/1757-1626-2-7905

**Published:** 2009-08-25

**Authors:** Joideep Phadnis, Paul S Trikha, David G Wood

**Affiliations:** 1 Department of Trauma and Orthopaedics, Epsom General HospitalDorking Road, Epsom, Surrey, KT18 7EGUK; 2 Department of Orthopaedics and Sports Medicine, North Sydney Orthopaedic and Sports Medicine Centre286 Pacific Highway, Crows Nest, Sydney, NSW 2065Australia

## Abstract

**Introduction:**

We report a case of isolated, unilateral avulsion of the vastus lateralis tendon from its insertion at the patella. This was diagnosed by magnetic resonance imaging, and underwent successful surgical repair.

**Case presentation:**

A healthy 32-year-old national level power lifter presented with an isolated avulsion of the vastus lateralis tendon. After a failed course of conservative therapy he underwent surgical repair and a graded physical therapy programme. One year later he returned to full training with no evidence of re-rupture.

**Conclusion:**

This is the first reported case of an isolated vastus lateralis avulsion. Our experience suggests that magnetic resonance imaging is invaluable in the diagnosis of this condition and that surgical repair provides a good outcome in high demand patients.

## Introduction

Quadriceps tendon rupture is a well recognized, debilitating condition, often requiring surgical intervention to restore normal knee function [1-5].

The vastus lateralis is one of the four muscles that make up the quadriceps mass. It arises from the inter-trochanteric line, base of the greater trochanter and lateral linea aspera and inserts into the superior-lateral pole of the patella as a distinct tendinous entity [6,7].

Quadriceps tendon rupture is thought to primarily manifest in tendons with a pre-existing degenerate ultrastructure. This is typically as a consequence of metabolic diseases such as chronic renal failure, systemic lupus erythematosus (SLE), and diabetes [1,3-5,8]. Increasing age, obesity and administration of systemic steroids have also been shown to have a strong association with rupture of the quadriceps tendon [2,4,5,9].

Quadriceps tendon rupture is uncommon and may be unilateral, bilateral, complete or partial [9-12]. Bilateral ruptures are rare and even more strongly associated with primary systemic diseases which affect tendon integrity [2,4]. Anatomically, rupture of the quadriceps tendon may occur in the musculotendinous or intratendinous regions but is most common at the osteotendinous junction [5]. It usually manifests as a result of rapid eccentric contraction of the quadriceps, with a flexed knee and fixed foot, although the mechanism of injury may be less severe in tendons with a more degenerate pre-existing ultrastructure [5].

We describe the first reported case of an isolated vastus lateralis avulsion which was suspected clinically, diagnosed by MRI and underwent surgical repair.

## Case presentation

A 32-year-old, Australian Caucasian man who was a national competing power lifter was referred to our specialist sports clinic 8 weeks after experiencing sudden intractable pain in his left knee, accompanied by a loud popping sound, whilst leg pressing 300 kg. Weight bearing and extension of his left leg were subsequently limited as a result of pain. Prior to attending our clinic, he had been managed non-operatively by his General Practitioner and a Physiotherapist, who had provided him with a knee brace for support. Despite this, he suffered continued pain and weakness, but denied any locking, giving way or instability.

**Figure 1.**
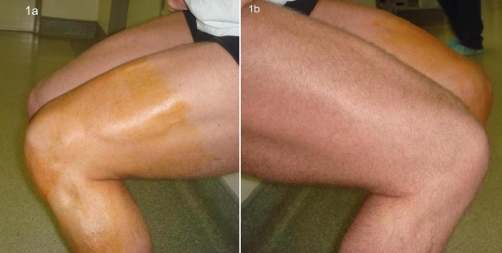
Squatting side views of both legs. **(a)** demonstrates retracted vastus lateralis muscle belly with void at insertion site, in comparison to normal right leg **(b)**.

He was a non-smoker, with no past medical problems and expressly denied using systemic steroids. He had not previously experienced any knee problems. Clinical examination revealed left quadriceps wasting in comparison to the contra-lateral leg with a palpable gap over the superior-lateral aspect of his patella. Knee flexion, in particular squatting, evoked pain and accentuated the gap. This also further demarcated the prominent, retracted vastus lateralis muscle belly (Figure 1). A normal range of active movement was retained; however power in extension was diminished in comparison with the other leg. There was no extension lag and no clinical evidence of ligamentous or meniscal pathology within the knee joint.

Blood tests showed no evidence of haematological or metabolic abnormality.

**Figure 2.**
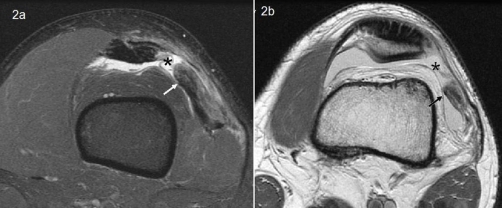
Axial fat suppressed image just above the patella (Figure 2a) and at the superior pole of the patella (Figure 2b) demonstrating complete tear (*) of the vastus lateralis. The oblique sagitally oriented component of the tear is propagating proximally at the interface between the Vastus Lateralis, Rectus Femoris and Vastus Intermedius. Arrow indicates the vastus lateralis tendon edge.

Anterior-posterior, lateral and skyline patella radiographs of the knee showed no diagnostic irregularity and no evidence of femoral trochlea dysplasia or variation in patella height or tilt. MRI demonstrated a complete and isolated avulsion of the vastus lateralis component of the quadriceps tendon in the osteotendinous region at its patella insertion (Figure 2). The patient was keen for operative intervention as he had already undertaken a course of unsuccessful conservative therapy and was anxious to expedite his return to training.

**Figure 3. (a), (b):**
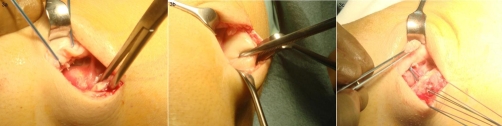
Demonstrate the proximal and distal ends of the avulsed Vastus Lateralis tendon at the point of its insertion on the patella. Note the healthy macroscopic appearance of the tissues. **(c)** Re-attachment of the avulsed tendon using suture anchors.

Intra-operative findings confirmed an avulsion of vastus lateralis with a residual stump of osteotendinous tissue still attached to the patella (Figure 3). The remaining quadriceps tendon was well attached and appeared macroscopically normal. Two Super Mitek anchors pre-loaded with Orthocord dual sutures (DePuy, California) were introduced to the patella gaining a firm hold. The tendon was then reattached to the patella using these anchors affording stable fixation (Figure 3). The primary repair was re-enforced by continuous soft tissue, Mason-Allen type absorbable sutures. Patella tracking and quadriceps tendon tension were compared intra-operatively to the contra-lateral knee and showed no variation. MRI scans taken two weeks after surgery demonstrated intact repair of the vastus lateralis tendon with in situ suture anchors (Figures 5 and 6).

**Figure 4.**
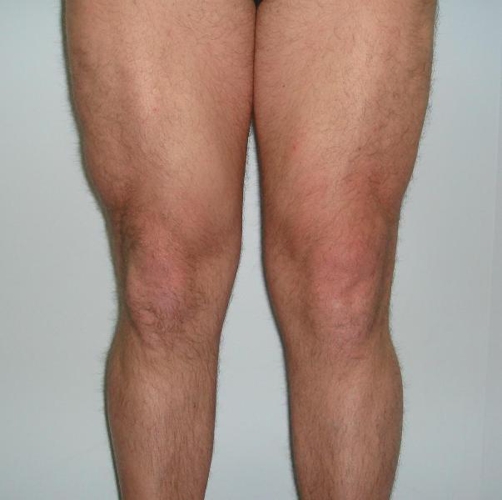
4 weeks post repair. Note the comparative left quadriceps wasting and well healed surgical scar.

Post operatively the knee was immobilized in an extension brace restricting movement from 0-45° for six weeks with protected weight bearing (Figure 4). At this point the brace was removed and a graded physical therapy program was commenced aiming for return to full training at six months. One year later, the patient returned to full, pain free training, but whilst leg pressing was only able to lift 70-80% of the previous weight he was pressing. Clinically there was no evidence of decreased power or range of movement, and the repair appeared intact.

**Figure 5.**
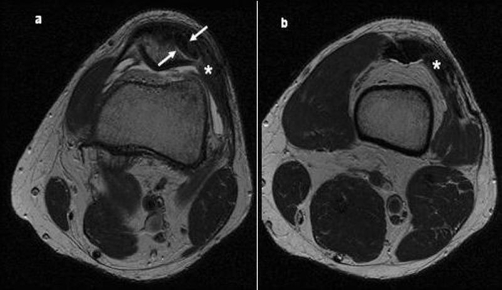
Axial T2 weighted image at the level of the superior pole of the patella (Figure 5a) and just above the patella (Figure 5b) two weeks post repair. 5a demonstrates the intact Vastus Lateralis tendon repair at the patellar insertion (asterisk) and more proximally (Figure 5b, asterisk) at the interface between the vastus lateralis, rectus femoris and vastus intermedius. Residual thickening and mild signal hyper intensity seen in relation to the repaired tendon indicates that scar remodeling is not yet complete. Note the metallic suture anchor in the superior pole of the patella (Figure 5a white arrows).

**Figure 6.**
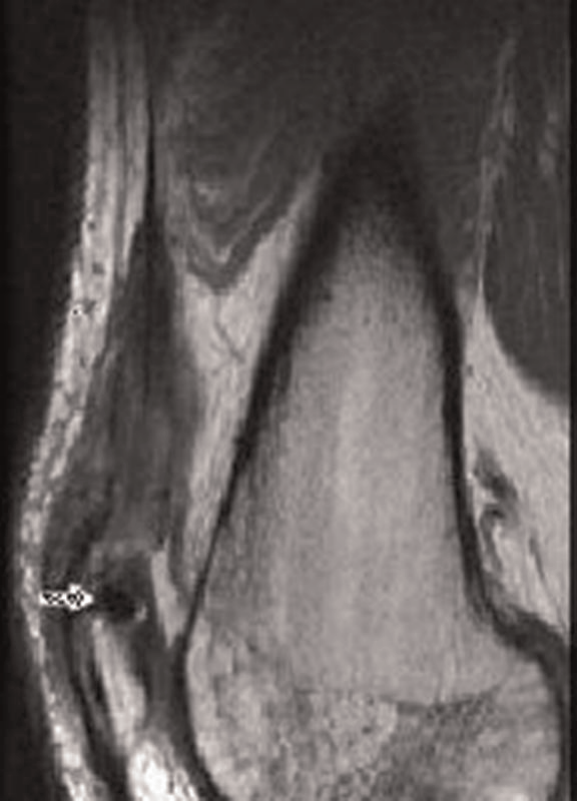
Sagitall image of the Vastus Lateralis tendon two weeks post repair. Demonstrates the metallic suture anchor in the proximal pole of the patella (white arrow) and post surgical scarring more proximally at the interface between the vastus lateralis, rectus femoris and vastus intermedius.

## Discussion

Clinical diagnosis of quadriceps tendon rupture is not always straightforward, and imaging is often required for confirmation [5,10]. Standard radiographs may show indirect signs of the presence of rupture, but are not generally helpful in management [2,5]. Sonography is useful, but magnetic resonance imaging (MRI) remains the gold standard because of its ability to clearly delineate partial tears and its consequent role in pre-operative planning [2,5,7,10].

Surgical repair is widely recommended for cases of complete rupture to prevent long term functional impairment [3,4,5,8,11-13]. Partial ruptures are those that do not involve the whole tendon mass and there is less consensus regarding the management of these tears. Conservative therapy is generally advised; however there is a role for surgery in high demand patients or those with failed conservative therapy [3,5]. Traditionally used surgical repair techniques have included direct suture repair; using drilled suture tunnels in the patella; the Scuderi technique for augmentation of direct repairs and the Codivilla lengthening technique used in shortened chronic tears [4,5,8]. More recently, fixation of the tendon with suture anchors on the patella combined with soft tissue reinforcement has been used with good results [11,13]. We chose to use suture anchors pre-loaded with Orthocord because being partially absorbable it would provide prolonged support to the tendon repair which would have to resist high volume dynamic loading as our patient recommenced training. In taking consent for surgery, we particularly highlighted the variation in evidence for repairing partial quadriceps tendon tears, and that to our knowledge; repair of such an injury had not been described in the medical literature.

While isolated vastus lateralis avulsion has not been described, Bikkina et al. [10] and Lewis et al. [9] both report cases of bilateral, complete, quadriceps tendon rupture in weight lifters undergoing surgical repair using drilled suture tunnels. However, in one case the patient was a long-term anabolic steroid user, and in both cases the patients were unable to return to their pre-injury level of training and suffered ongoing symptoms. Kayali et al. [12] reported a case of bilateral, atraumatic Quadriceps tendon rupture in a patient undergoing haemodyalisis, repaired successfully with drilled suture tunnels but with the additional augmentation of a quadriceps tendon flap. Shanmugam et al. [14] described rupture of the quadriceps tendon in a patient with previous patellectomy repaired by end-to-end sutures. It must be noted that in these two cases, the quadriceps tendon was at risk of rupture due to metabolic disease in the first case and mechanical imbalance in the second. In contrast, we speculate that by attempting to lift such a heavy load in a rapid, eccentric manner, our patient generated sufficient force to avulse a completely healthy tendon.

Our case illustrates that partial quadriceps tendon rupture may occur purely due to excessive mechanical forces and in this scenario appears to yield good results with surgical repair even in high demand individuals.

## Conclusion

This is the first reported case of an isolated avulsion of the vastus lateralis tendon. For an elite athlete this is a potentially debilitating condition and may be overlooked by clinicians because of the integrity of the vast proportion of the Quadriceps tendon. This case report raises awareness of this condition and indicates that surgical repair is successful in expediting recovery in high demand individuals.

## Abbreviations

MRI: magnetic resonance imaging
SLE: systemic lupus erythematosus
